# The Impact of the COVID-19 Pandemic on eHealth Use in the Daily Practice and Life of Dutch-Speaking General Practitioners in Belgium: Qualitative Study With Semistructured Interviews

**DOI:** 10.2196/41847

**Published:** 2022-11-28

**Authors:** Jan Ismail Yagiz, Geert Goderis

**Affiliations:** 1 Leuven Institute for Healthcare Policy Medical Faculty KU Leuven Leuven Belgium

**Keywords:** COVID-19, impact, eHealth, GPs, Flemish, practice, qualitative study, semistructured interviews

## Abstract

**Background:**

The COVID-19 crisis has led to rapid and far-reaching changes in digital health care, but little is known about what, why, and how changes occurred in eHealth use in Flemish general practice during the pandemic.

**Objective:**

This study aims to understand how general practitioners (GPs) perceive and evaluate eHealth solutions and their eHealth experience during the COVID-19 pandemic.

**Methods:**

This qualitative study was conducted using in-depth 1-on-1 semistructured interviews with the help of an interview guide. Several areas were identified beforehand to help assess the impact of the COVID-19 pandemic: perceptions of digital technologies in GP practices; changes in the use of these technologies during and after the COVID-19 pandemic; GPs’ adaptation to digitalization, benefits, risks, and challenges of eHealth; GPs motivations to change practice; and future perspectives on eHealth. In this study, purposive sampling and snowballing methods were used. Between October 2021 and April 2022, we interviewed 15 Dutch-speaking GPs in the Flemish region via the Zoom online conferencing tool.

**Results:**

GPs indicated that eHealth was used more frequently during the COVID-19 pandemic than before, a change that helped them reduce their workload, enabling greater accessibility to health care services and the complementary use of digital and physical consultations. Our findings suggest that physicians underwent a significant cognitive shift in their perceptions, causing them to be more open and prepared to adopt eHealth solutions. However, there remains significant doubt and uncertainty about digital literacy for certain groups, privacy, data security, reimbursement, and the burden of technical information and communication technologies (ICT) issues.

**Conclusions:**

The COVID-19 pandemic seems to have been a turning point for eHealth by Flemish GPs. eHealth is an essential complementary health care service that can reduce pressure on health care as well as increase health care accessibility. Sensitive aspects, such as privacy, data security, digital literacy, reimbursement, and the burden of technical ICT issues, are particularly emphasized. With our results, we can offer recommendations to health IT policymakers and developers that will help maintain the continuity of eHealth solutions beyond the COVID-19 pandemic, considering the expectations and sensitivities presented in the study.

## Introduction

eHealth refers to tools that use information and communication technologies (ICT) to prevent, diagnose, treat, and monitor health-related problems. The term “eHealth” is inclusive, and what falls under eHealth is also constantly changing under technological developments, such as mobile health (mHealth), telemonitoring, telemedicine, cloud platforms for data storage, and artificial intelligence (AI). The term “mHealth” refers to the use of mobile communication and devices that provide care or improve health outcomes, for example, with the help of an app on smartphones [1]. Telemonitoring is described as daily remote, wireless, and ambulatory monitoring of various medical and technical data and parameters via sensors, cameras, and devices implanted in the patient or placed on the body or in the patient's clothing, such as blood pressure, weight, heart rate, and body temperature [2]. The term “telemedicine” relates to health care services that permit patients to receive care in their everyday life and overpass the distance between health care professionals and patients through ICT [3].

eHealth solutions are represented as complex interventions, as several interacting components pose some additional problems for raters and already theoretical and methodological issues [4]. However, over the past decade, the growing use of eHealth has increased pressure on health care [5,6], playing an increasingly important role in the sustainability of future health care systems and increasing passion in patient empowerment [7,8]. An aging population, rising chronic diseases, and the COVID-19 pandemic are increasing pressure on health care [9-12]. Therefore, innovations, such as eHealth, are needed to maintain the accessibility and quality of care [13-16]. Meanwhile, digital health technologies have greatly accelerated patient engagement [17-20]. In line with these developments, medical institutions have intensively integrated eHealth into traditional face-to-face counseling [21]. The combination of eHealth and face-to-face consultation can be defined as hybrid health care [22,23]. Sometime after the COVID-19 pandemic, it was shown that the pandemic has accelerated the implementation of eHealth solutions, and even though it is a crisis situation, many health care providers and institutions have quickly embraced digital medicine. Adopting more digital health is a phase with the potential to improve the quality of care [24,25].

General practitioners (GPs) are an essential part of primary health care in Belgium's health system. They usually work in solo, dual, or group practices, and their main tasks include preventive care, diagnosis, and treatment for a wide range of health problems. Since 2007, digitization systems have been used in health care in Belgium [26].

According to a Belgian 2019 study, the adoption of eHealth by GPs in Belgium—as in other countries—remains modest [27]. These results are in line with other studies showing that the health care sector is a laggard in adopting digital services [28]. According to these studies, several factors play an active role in the sector's slowness to adopt digital practices, such as the strict regulations of the sector, the sensitivity surrounding personally identifiable information, the resistance of health care providers to digital apps, the lack of prioritization of the patient experience, and the cost of investment [29,30].

The first COVID-19 death in Belgium occurred on March 10, 2020, and the first quarantine period was imposed on the population from March 14 to May 5, 2020, to prevent the spread of the COVID-19 virus [31,32]. Rapid and far-reaching changes have been made so that health care providers can ensure continuity of care while minimizing the risk of the virus spreading. Digital health tools, such as social follow-up, texting, getting photos from patients, and e-consultations, have been used more than ever [33]. In Belgium, temporary remote consultations (by phone or video calls) were introduced. According to the COVID-19 monitoring report of the National Institute for Health and Disability Insurance (RIZIV), 3.8 million consultations were billed between March and May 2020, and these consultations were performed mainly by GPs. Teleconsultations were commonly used to obtain prescriptions, follow up on a chronic or existing condition, deal with new coronavirus-related complaints, and discuss a sickness report or a new complaint unrelated to coronavirus [34].

Although other studies have previously shown that the COVID-19 pandemic significantly impacted GPs’ use of eHealth, little is known about the impact of the COVID-19 crisis on the daily use of eHealth technologies among GPs in the Flemish region of Belgium. In our study, we investigated the following research questions: What has changed in the mind and practice of GPs toward the use of eHealth as a result of the COVID-19 crisis? What other changes were experienced by GPs during this period, why, and how? Moreover, we aimed to gain deep insight into the perceived impact of the crisis.

This paper describes the GPs’ perception, appreciation, and use of various eHealth tools during the pandemic; their expectations; concerns regarding eHealth use; the perceived impact of the COVID-19 pandemic on eHealth use; and perceived barriers, enablers, potential gains, and success points in eHealth use.

## Methods

### Study Design and Population

**Figure 1:**
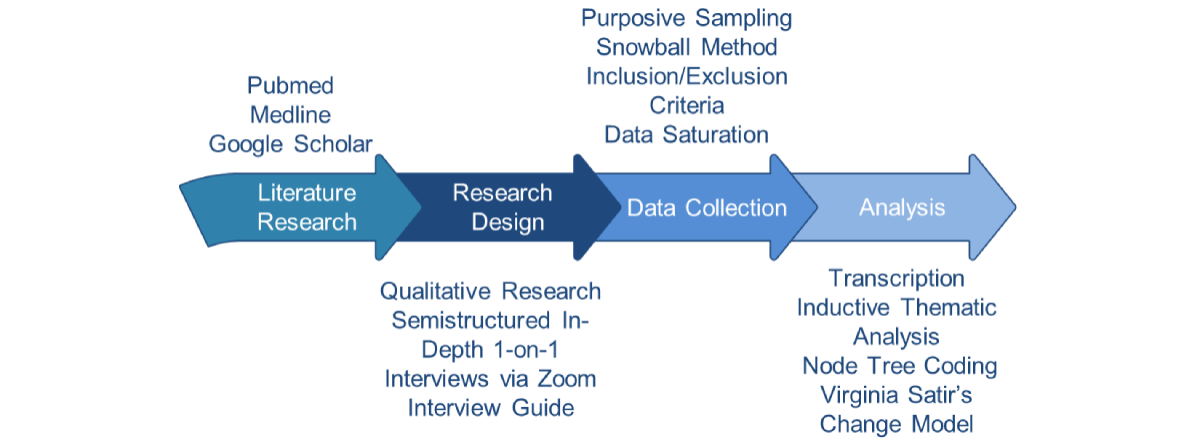
Study design.

We conducted qualitative research using semistructured interviews of 15 Dutch-speaking GPs in Belgium. One-on-one interviews were chosen as the research design, which allowed us to obtain in-depth information from the respondents. Purposive sampling and the snowball method were used during the study. The study population was assumed to be around 20-30 GPs, but when data saturation provided enough knowledge for a deep understanding of the subject, the study finished before the targeted population numbers were reached. The study was conducted between October 2021 and April 2022. The study design is shown in Figure 1.

### Interview Procedures, Data Collection, and Analysis

**Figure 2:**
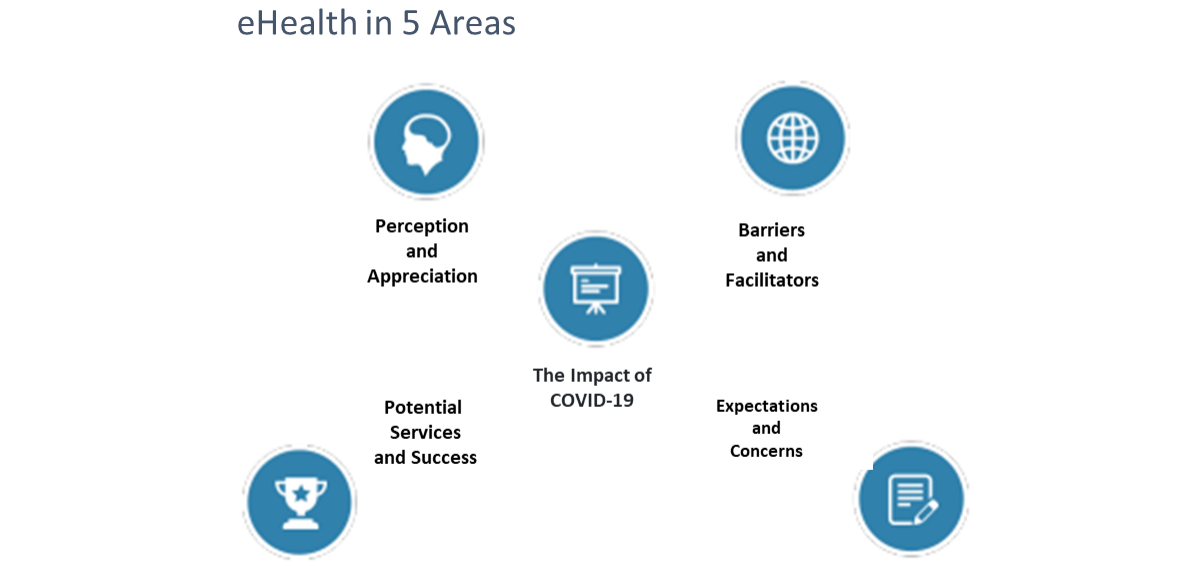
Defined themes.

Due to the COVID-19 pandemic, interviews were scheduled and conducted through the Zoom online videoconferencing tool. The interview guide (Multimedia Appendix 1) was developed in close collaboration with the promotor. Before the interviews, informed consent was collected via email from the interviewees. The subinvestigator conducted the semistructured interviews using the following thematic blocks: demographics; the perception and appreciation of digital technologies in GP practices; changes in the use of these technologies during and after the COVID-19 pandemic; the adaptation of GPs to digitalization; eHealth's benefits, risks, and challenges; and motivations for and future perspectives on eHealth. The interviews lasted approximately 30 minutes. All interviews were recorded, transcribed verbatim, and analyzed thematically using Microsoft Excel 2010. Braun and Clarke's [35] 6-step guide was used. Notes were grouped into 5 domains (with themes and subthemes): the perception and appreciation of eHealth, the impact of COVID-19 on eHealth use, the barriers to and facilitators of eHealth use, the potential services and success points of eHealth, and concerns and expectations for the future (see Figure 2). All quotes were translated from Dutch to English, considered representative, and are reported in the Results section.

All data were treated confidentially and pseudonymized with due care during the project. Depending on our research questions, the recorded interviews were only watched and listened to and typed out by the researcher supervised by the promotor. The interviews were transcribed in an anonymous manner. The sound recordings and transcripts were recorded in a safe and secure way, and these files were saved on a personal computer that is password-protected. The study was conducted on a volunteer basis, and there was no compensation given.

### Ethical Considerations

Ethical committee approval and informed consent letters were asked for and obtained beforehand from the KU Leuven Ethics Committee (reference MP016788, July 20, 2021).

## Results

### Characteristics of Study Participants

We interviewed 15 GPs from 5 different regions of Flanders: Antwerp (n=7, 47%), Limburg (n=3, 20%), Flemish Brabant (n=2, 13%), East Flanders (n=2, 13%), and West Flanders (n=1, 7%). Of the 15 interviewees, 10 (67%) reported practicing medicine for 0-20 years and 5 (33%) reported practicing medicine for more than 20 years. On average, they had 14.8 years of experience in primary care, from 3 to 40 years. Most interviewees reported working in a group (n=7, 47%) or alone (n=5, 33%). The gender distribution was 8 (53%) males and 7 (47%) females. The demographic characteristics of the participants are shown in Table 1.

Based on the thematic analysis of the interviews, we collected all the data on 5 important domains with subdomains, as shown in Tables 2-6.

**Table 1:** Demographics of participants (N=15).

Experience (years)	Solo practice, n (%)	Duo practice, n (%)	Group practice, n (%)	Community, n (%)	Total GPs^a^, n (%)
<5	0	0	2 (13): M^b^, Antwerp, GP7 and GP8	0	2 (13)
5-10	1 (7): F^c^, Antwerp, GP9	0	2 (13): M, East Flanders and Antwerp, GP10 and GP11 2 (13): F, Antwerp, GP5 and GP6	2 (13): M, Brussels, GP1 and GP2	7 (47)
10-20	0	1 (7): F, Limburg, GP14	0	0	1 (7)
>20	2 (13): F, Limburg, GP3 and GP4 2 (13): M, West Flanders, GP13; F, Antwerp, GP12	0	1 (7): M, East Flanders GP15	0	5 (33)

^a^GP: general practitioner.

^b^M: male.

^c^F: female.

**Table 2:** Themes and subthemes of the “impact of the COVID-19 pandemic on eHealth use” domain.

Themes and subthemes			Quotes from the interviews
**Changes and shifts in service**			
	There is greater eHealth uptake.	“COVID created an extra training for us, especially for older GP^a^ generations who were against the digitalization but came to understand the benefits of eHealth.” [GP2]	
	GPs reduce strain with eHealth.	“Teleconsultation is not a pleasant thing, but it was an important tool in the intensity of the pandemic.” [GP3]	
**Teleconsultations**			
	GPs provide triage for suspicious infections.	“We did triage for COVID infection via teleconsultations.” [GP5]	
**Workload**			
	GPs’ workload increased and sped up the process of digitalization.	“COVID sped up and also increased our work via digitalization. It also changed our way of working.” [GP6]	
**Digital administrator**			
	GPs provide e-certificates, e-receipts, and reimbursement papers.	“COVID has changed the way we work and boosted digital solutions.” [GP10]	
**Extra investment in IT solutions**			
	New investments increase in eHealth, mHealth^b^, and video consults.	“We adopted an application on the mobile phone so that we could also work from home.“ [GP7]	

^a^GP: general practitioner.

^b^mHealth: mobile health.

**Table 3:** Themes and subthemes of the “eHealth perception and appreciation” domain.

Themes and subthemes		Quotes from the interviews
**eHealth perception**		
	eHealth is a digital solution for GP^a^ work.	“eHealth is a portal to digital to use all the capabilities of information, EMD^b^, tele-consult, medication…” [GP1] “Digital solutions increased the quality of and communication in health care.” [GP8]
	The future is digital.	“Digital apps will be used more because GPs are retired, and the time spent on physical contact will be less than now.” [GP4]
**eHealth appreciation**		
	eHealth has a lot of advantages.	“There are many positive things.” [GP1] “Our clinic is 90% digital now.” [GP6] “I find it very good to have. I hope that will get better, be adopted more quickly, and implemented at a faster pace. I found it very slow, I mean, the evolution over the two years.” [GP13]

^a^GP: general practitioner.

^b^EMD: Electronic Medical Dossier.

**Table 4:** Themes and subthemes of the “potential services and success points” domain.

Themes and subthemes		Quotes from the interviews
**Potential services**		
	GPs^a^ are prepared for the unknown.	“I think we are more ready for the unknown…the crisis made us stronger and able to provide health care in difficult situations.” [GP10]
	GPs have more digital literacy.	“Software is new, and I am not fully integrated with the software. I need more training so that I can use it more efficiently.” [GP14]
	GPs have sight for mHealth and video consultations in the future.	“The role of the GP will be changed to include controlling data from applications. We could follow up closely chronic diseases with the applications, especially with chronic illnesses such as heart failure” [GP15] “I want to implement video consultations in the future, especially so that I can see the patient.” [GP9]
**Success points**		
	There is increased accessibility.	“I am in urgency mode for the patients, reachable 24/7.” [GP12] “Quick connecting with people to monitor them at home, especially older people” [GP3]
	GPs are aware of the mental shift to remote work.	“One of the most important things we learned from the coronavirus crisis is that eHealth solutions shifted our mindset from classical role of the physician to an integrated role using more remote solutions.” [GP11]
	Physician-patient satisfaction with telemedicine	”In general, the patients are satisfied with the speed of work. They do not wait long and do not come here to take medications.“ [GP4]

^a^GP: general practitioner.

**Table 5:** Themes and subthemes of the “barriers and facilitators to using eHealth” domain.

Themes and subthemes		Quotes from the interviews
**Barriers**		
	There is an ICT^a^ burden.	“Lack of IT support to solve the problems such as older software or blockages.” [GP^b^4]
	Digital literacy is needed.	“To use the digital platforms, you need to have an educated population.” [GP1]
**Facilitators**		
	COVID-19 pandemic measures	“Due to the measures, the disallowing of physical contact made us facilitate creative solutions, such as using more technology and remote options.” [GP5]
	Government and European Union (EU) policies	“Remuneration for telehealth was useful to stimulate complementary remote and physical care.” [GP9]
	Patient’s role	“The patient is central now. We have to listen to them, and most patients are comfortable and are doing well with the technology and happy to use it.” [GP12]

^a^ICT: information and communication technologies.

^b^GP: general practitioner.

**Table 6:** Themes and subthemes of the “future expectations and concerns” domain.

Themes and subthemes			Quotes from the interviews
**Expectations**			
	GPs^a^ were seeking for integrated first and second service lines.	“More stable eHealth, technically strong and more integrated solutions. Currently, digital integration between the 1 and 2 service lines is terrible.” [GP13]	
	GPs expect more efficient IT solutions.	“I would like to see more user-friendly solutions for all population levels, as well as more education on digital eHealth solutions.” [GP10]	
	GPs demand remuneration for telemedicine services.	“We would like to have further remuneration for teleconsultations.” [GP9]	
**Concerns**			
	GPs are aware of safety concerns related to data.	“We often use e-mails, text messaging, and WhatsApp. These are not fully safe, not fully protected. These must be protected from cyberattacks.” [GP8]	
	GPs are concerned about fragile groups.	“There are subgroups of patients who are not aware of or capable enough to use eHealth tools.” [GP15]	
	GPs have concerns about losing physical contact and follow-ups.	“I do not want to lose my physical contact with the patient…Why? Problems with follow-up. Why? Because even though it is a prescription, I am doing more than that. Are there any other issues to follow up on? For example, screening for colorectal cancer, mammography, vaccinations, blood pressure measurements, advice, conversations with the patient.” [GP1]	

^a^GP: general practitioner.

### Domain 1: Impact of the COVID-19 Pandemic on eHealth

#### Changes and Shifts in Service

According to the GPs surveyed, eHealth use showed a tremendous difference before and after the COVID-19 pandemic. All the participants agreed on the differentiation and increased use of eHealth tools.

Before the coronavirus crisis, they noted that they were using eHealth frameworks infrequently or in a limited number of cases, and they used phone calls and emails mainly for simple investigations. However, they had to find new arrangements during the pandemic due to limited in-person contact. Teleconsultations and eHealth apps, such as e-receipts, e-certificates, digital reimbursement, and online appointment modules, were widely used.

They also stated that a few apps that had been in use before the pandemic worked exceptionally well during the pandemic, such as e-receipts. They generally summarized the impact of the coronavirus crisis on eHealth use in 3 domains: an expanded uptake of eHealth in their practice, decreased strain on work with eHealth arrangements, and differentiation of the use of eHealth solutions.

##### Increased eHealth Uptake

Digitalization increased after COVID in our practice from 40% to 75%...The quality of digitalization is also better, and it is easier than before.

GP14

We were using telephone contacts with our initiative for talking about patient results before COVID, but now we use it for teleconsultations, triage, and prescriptions.

GP15

##### GPs Reduced Their Work Strain With eHealth

COVID increased the use of e-certificates, e-receipts, and other online solutions that helped us and patients to get out of this crisis in a healthy way.

GP10

#### Workload

All the interviewees agreed on differentiation in workload due to the COVID-19 pandemic. They said their work increased, was boosted, and sped up with the impact of the COVID-19 crisis on eHealth use.

Meaningful feedback from GP1 was:

COVID increased our workload, sped up digital solutions, differentiated the use of tools, and implemented teleconsultations to solve urgent problems.

GP1

#### Teleconsultations: The New Way of Working

All the GPs have implemented teleconsultations since the start of the pandemic. The GPs said they used teleconsultations as a complementary tool almost daily in their practices. They blocked extra hours for teleconsultations between physical consultations. They found it critical for continuity of care, and they used it as an exit strategy during the enormous workload of the pandemic. Teleconsultations were used for working safely, triaging, and following patients.

Teleconsultation is not a pleasant thing, but it was a way for us to overcome the intensity of the pandemic.

GP3

##### Creating a Safe Working Environment and Triaging via Teleconsultations

Via teleconsultations, we protected ourselves from COVID infection, we could work safely, and we guaranteed the continuity of care during lockdowns.

GP12

#### GP as a Digital Administrator

All the interviewees noted general displeasure about digital administration for different reasons. They all pointed out that the pandemic brought many extra administration duties at the expense of patient care.

The impact of COVID was huge. Suddenly, we had e-certificates that we had to deliver to patients, but there was the practical problem that the digital signature was not accepted, so every patient had to come to us again, but they could not because they had to stay home…So, we had to sign certificates for these people, which is absurd; if someone had a positive test and got a digital result, why should I have to write a homestay certificate again?

GP13

#### Increased Investments in IT

During the pandemic, GPs were aware of eHealth benefits. They described how they tended to invest in IT solutions from the start of the pandemic and noted that they searched for and invested in more stable software, online modules, and providers.

GP12 described how the COVID-19 pandemic boosted the investment in her practice:

I had been thinking about switching to an online appointment program for many years. The COVID-19 pandemic led me to invest in this program with an extra telemedicine module.

GP12

GP7, who worked in a group practice, added that with the new investments, all the colleagues worked more flexibly and remotely:

Now, everyone at the practice has laptops with new software that we can take home so that we have access to our medical records at home.

GP7

### Domain 2: Perception and Appreciation of eHealth

#### Digital Communication Tool

Across multiple interviews, all the GPs defined eHealth as a digital hub of instruments and administrations that provides different health-related tools. The primary eHealth solutions were private software for patient dossiers (eg, Care-connect), online appointment modules, online secretary tools, telemodules, and mHealth apps (eg, the Collaborative Care Platform [CoZo], e-receipts, e-certificates).

The interviewees stated that eHealth apps were critical during the COVID-19 pandemic and that their perception of eHealth, which they previously associated with ICT problems, has changed positively due to the pandemic because they use this system more efficiently.

Digital solutions increased the quality and continuity of health care.

GP1

GP2 shared vital feedback about how the old GP generations appreciated eHealth during the COVID-19 pandemic:

COVID gave us extra training, especially older GP generations who were against the digitalization. It allowed them to understand the benefits of it because before that; they were not using any digital solutions even though we had them.

GP2

#### The Future Is Digital

GPs stated that they work predominantly in the digital environment and that eHealth is the future.

The more, the better, I am happy.

GP13

This is an inevitable future.

GP15

#### Advantages of eHealth

GPs noted that providing primary care via eHealth gave them immense satisfaction in their work. The participants noted 3 main advantages of eHealth. First, eHealth collected all data easily, quickly, and efficiently. Second, it saved time. Third, it sped up their work, especially in urgent conditions.

Thanks to eHealth, I save time and I spend this extra time with my family. Before I had to take all the documents home, which was extra work, but now, it is effortless.

GP14

GP1 added that eHealth has ”a lot of positive things…easy to find, collect, and deliver data, easy to work paperless, easy to send medicine, easy to work quickly, easy to send invoices…“

#### Disadvantages of eHealth

All the GPs added that there were some disadvantages that they had to overcome, such as technical problems, sitting for too long in front of the computer, a lack of physical contact with the patients, and being perceived as a ”24–7 online doctor.“

Mmm…I think we see more patients in a short time and have shorter contact with the patient, which is an advantage, but at the same time also a disadvantage because the patient has the idea that we are always accessible and he can say, “I have a skin problem, I will take a picture of it and forward it to the GP by e-mail,” and he thinks that he will immediately get an answer. Unfortunately, it usually does not work, so a bit of a fine-tuning is still needed, not only for us but also for the patients, to figure out how we should deal with that.

GP12

### Potential Services and Success Points Related to eHealth Use

The GPs noted several possible services and success points during the pandemic regarding eHealth use. First, they all stated that the COVID-19 pandemic trained them to cope with the unknown. They were forced to deliver care during the lockdowns and had to find more innovative care delivery methods.

#### Potential Services

##### Preparedness for the Unknown

The interviewees acknowledged that eHealth use and new experiences prepared them for other possible pandemics or health crashes. Some of them described how they implemented their initiatives.

After the first shock, we were urged to deliver care only for emergencies and COVID infections. All other things were canceled. We created a common Excel document with our patients' data categorized with colors (eg, red meant urgent, yellow meant less urgent, green meant solved), and we had to check that frequently. This was our adaptation to keep track of our patients. In addition, we moved physical consultations with COVID-like illnesses to very early in the morning and had to disinfect all the practice rooms after. These were different solutions we had to find. We met continuously with other colleagues to learn and share new things.

GP5

##### Digital Literacy

All the participants said they were busy improving their digital literacy to use eHealth effectively during the lockdowns.

As a practice, we asked for extra pieces of training and workshops from the providers.

GP14

##### mHealth and Video Consultations

The responders indicated that they used mHealth apps and video consultations after telephone consultations.

I do not use applications to follow the patients, but I think that would be interesting. For example, diabetes follow-ups. This would be exciting if people take a test at home and integrate the results with their medical files. I also think there are applications for it.

GP6

A few of them tried to use video consultations for selected patients. They all acknowledged that a stable internet connection and technical competency were needed for successful telehealth practices:

The next step we were thinking of was implementing video consultations. We saw it during a congress in the United States, and we thought about it before the COVID-19 pandemic, but now we are busy with investment for that.

GP2

#### Success Points

##### Increased Accessibility

The convenience of remote care made it easier for patients to reach doctors in the first months of the COVID-19 pandemic when the quarantine instructions were applied. Using eHealth solutions allowed GPs to coordinate primary health care and safely communicate with patients.

Using eHealth is helpful because people can have greater access to care during the pandemic even outside working hours. We are available at night and on the weekends to provide the urgent needs. eHealth solution makes people reach our services and increase accessibility, which is critical during the pandemic.

GP8

##### The Mental Shift to Remote Work

GPs collaborated through physical and remote work and achieved good results from their remote work. A mind shift occurred during remote consultations. This mind shift was 1 of the vital breaking points to fastening eHealth solutions' uptake. The GPs said they had to find answers to solve problems urgently and remotely. In this way, they changed their work and this allowed them to collaborate physically and via teleconsultations.

The COVID-19 pandemic forced us to solve problems remotely, which required a mental shift to combine physical and teleconsultations.

GP10

One of the most important things we learned from the COVID crisis is that eHealth solutions shifted our mindset…from relying on the traditional doctor's role to considering an integrated role using more remote solutions.

GP11

##### Physician-Patient Satisfaction With Telemedicine

As doctor-patient relationships evolved over the past decades, communication became more diverse during the pandemic. The satisfaction of patients became more crucial than ever according to the surveyed GPs. The interviewees added that young adults used all the digital technologies efficiently, and adaptation to eHealth services during the pandemic was high. Using eHealth allowed patients not to replace their selves, to find quick solutions remotely, and to be satisfied with the health service.

This was a comfort for the patient, no transport, no parking, no time wasted on coming to the doctor.

GP4

Most patients are comfortable and are doing well with eHealth solutions. They are happy to use them.

GP12

### Domain 4: Barriers to and Facilitators of eHealth

#### Barriers

According to GPs, there were 2 critical barriers to eHealth uptake. Although eHealth barriers diminished compared with previous studies, the ICT burden and patients’ digital literacy remained.

##### ICT Burden

eHealth information does not work on Monday, works on Tuesday, Wednesday, Thursday, and then stops working on Friday. This is such a long weekend, and things are not reachable, insurance and citizen numbers cannot be seen…then, we are blocked.

GP4

##### Digital Literacy

The responders commented on another significant barrier: patients' digital literacy. They found that this was critical for providing equal access to services during the pandemic. The patients who did not understand how to use digital health tools could not access those tools in their language, which disadvantaged them.

If you work in the suburbs of Brussels, you know that some populations cannot speak Dutch or French. They do not understand the invitation briefs from the government, and they come to ask what these papers say. Sometimes, the language used in official papers is very complex, not simple. These groups are also not eligible to use digital solutions. Education of these fragile groups is essential because they feel like they are outside of the community.

GP1

#### Facilitators

The GPs in our study also identified 3 main domains as facilitators to the uptake of eHealth. The biggest was COVID-19 measures, followed by government and European Union (EU) policies and the patients' active role during the pandemic.

##### COVID-19 Pandemic Measures

The COVID-19 measures applied for preventing the spread of the virus also reduced access to care. According to GPs, measures were a change moment for transforming ideas and discovering the benefits of eHealth solutions.

We had to find a solution to maintain care, which was difficult because everything was canceled...There was no more physical contact because of the measures, which forced us to devise creative solutions, use more technology, and get used to it…

GP5

##### Government and EU Policies

Remuneration for teleservices from the government and developed digital apps to provide PCR (polymerase chain reaction) tests and e-certificates accelerated the uptake for eHealth according to GPs.

We grew more accustomed to eHealth tools through providing for our patients by creating and sending PCR tests, e-certificates, e-receipts, and reimbursement papers.

GP7

##### The Role of the Patient

Patient demand for telemedicine outstripped the ability of health care providers to supply it. As our interviewees noted, there was considerable satisfaction on the part of the patient about reaching their doctor quickly and getting solutions during the pandemic, which stimulated greater eHealth uptake in health care.

Communication with the patient was online, quick, and necessary…They were more satisfied than us, I think. Patients using these tools efficiently definitely increased our adaptation to eHealth.

GP11

### Domain 5: Future Expectations and Concerns

#### Expectations

All the GPs were satisfied with the information circle between the first line of medical services, but they noted that there was no efficient information flow between the first and the second lines.

##### More Integrated First and Second Service Lines

Responders emphasized that they also had difficulties transferring information to the second and third medicine lines. They complained about a lack of integration between health care services in eHealth.

Every hospital has a different eHealth system. In 2022, this cannot happen.

GP13

We are working on the same patient, but there is a lot of missing data between the service lines. What I do cannot be seen by the specialist…

GP11

##### More Efficient IT Solutions

Another issue was stable IT support. Although IT support has significantly improved since the beginning of the COVID-19 pandemic, almost all participants declared that they expected more efficient IT solutions.

Stable, technically strong, and well-integrated IT solutions are expected in the future.

GP4

##### Remuneration for Telemedicine Services

All the interviewees agreed that receiving reimbursement was beneficial during the pandemic. One physician added that she and her colleagues were upset about the reimbursement department's decision regarding the cancellation of reimbursement for teleservices.

I am despondent about this decision. Remuneration for teleservices was first decreased and then canceled. We are busy collecting signatures to get back the teleconsultation reimbursement. I think this is very beneficial for us now and in the future. Remuneration for teleconsultations must be well regulated if the policymakers want us to cope with unknown circumstances…

GP9

#### Concerns

The interviewees were worried about some issues. The main concerns were the security of patients' data and fragile groups who were not eligible to use digital solutions and the loss of in-person contact.

##### Awareness of Data Security

First, the increase in digitalization made the GPs concerned about data regulation and safety. During the pandemic, there was an extensive unprotected circulation of patient data on different digital platforms. Emails, text messages, and WhatsApp messages were not protected enough according to GPs.

One thing we mostly forget is that the patients' data circulate on unprotected platforms like emails and WhatsApp...I find it personally very dangerous and open to cyber-attacks.

GP8

##### Fragile Groups

Second, they were sensitive to the patients with socioeconomic problems or language barriers and subgroups that were not eligible to use digital solutions. The GPs mentioned that fewer controls and in-person contact would probably lead to substantial health problems for these people. They expected to experience more distress from COVID-19 and the lockdowns for these fragile groups.

Cultural differences and language barriers were the main reasons patients did not adopt eHealth tools.

GP13

Some people were unaware of their health problems, and others were not eligible to use digital systems and needed in-person visits. These people are fragile, and access to health care could be more difficult if we only use digital platforms.

GP1

##### Losing In-Person Contact and Follow-Ups

Even though the shift from in-person to remote care during the COVID-19 pandemic was supposedly advantageous for GPs, there was a big concern about losing in-person contact with the patients. Some added that they deliberately minimized teleconsultations in order to avoid losing this contact with the patients, and others asked patients to come to the clinic after a few digital consultations.

We do now have a limit of 3 months for e-prescriptions. After that, they have to come physically to see us. We are limiting teleconsultations. Things are now returning to normal. We do not want to lose in-person contact with the patients because we are responsible for the quality of care.

GP2

##### Only Teleconsultations Are Not Enough

The GPs noticed that although teleconsultations were critical for continuity of care and used as an exit strategy during the enormous workload of the pandemic, pure digital consultations are unrealistic and cannot replace natural, physical interaction with the patient. Some GPs added that fully digital solutions are not enough to provide holistic care.

You cannot do everything digital…Some patients need physical contact. Others must be followed in-person contact because they are not fully ready to control their health and are unaware of what is going on…

GP13

We are holistic health care providers; we evaluate all the aspects of the patient, physiological, social, physical…Furthermore, unfortunately, in teleconsultation, we only make up for what it lacks. Therefore, we always say we leave the door open for in-person visits.

GP15

### Implementing Results on Virginia Satir's Model

**Figure 3:**
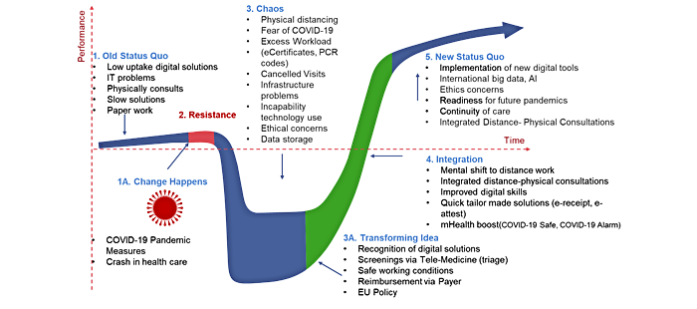
Adapted from Virginia Satir's change model [40]. AI: artificial intelligence; EU: European Union. PCR: polymerase chain reaction.

Why this eHealth uptake rapidly changed after years of slowness in adopting digital practices and why and how the COVID-19 pandemic became a tipping point are not easy questions to answer, because health care organizations are complex. It is not always possible to predict changes or the effects of interventions, because of the significant, interdependent, hierarchical, top-down, and fragmented characteristics of health care organizations [36,37]. Therefore, to implement a change in complex health care contexts, change management methodologies are often used as guiding principles [38,39]. In this study, we used Virginia Satir's change model to understand this change [40]; see Figure 3. This model was created by Virginia Satir, a family therapist, to aid individuals or organizations recover the way they deal with noteworthy, unpredicted change. Using this model in health care may help explain the changes because it includes hierarchical and growth models. The hierarchical model attempts to simplify life and allows for a complicated structure of layered responsibilities, whereas the growth model is geared toward the complexity of human interactions [41]. This model includes 5 stages: old status quo, resistance, chaos, integration, and new status quo.

In the old status quo phase, we can see how things were shortly before we realized the significant and disruptive change. Before the COVID-19 pandemic, eHealth uptake was low due to different barriers. There was less investment in eHealth services, and eHealth was perceived as time-consuming. Physical contact with patients was the cornerstone of care.

The resistance phase describes resistance to the foreign element that disturbs the comfort of the status quo. In this case, the pandemic caused a significant shock to the health care system. Continuity of care was disrupted, and there was a service shift to critical COVID-19–related issues. Teleservices were used extensively but were insufficient due to the loss of nonverbal language. Concerns were raised about the continuity of regular care from GPs.

In the chaos and idea transformation phase, the foreign element gains critical weight and changes work. The old way of working is not adopted. Performance declines, and a sense of urgency emerges—feelings of stress, confusion, vulnerability, fear, and panic arise. Nevertheless, these feelings can generate new ideas, which will be a path to transformation. Crucial to this stage is to see how the external element can benefit through a transformative idea. Trying new ways of working and acquiring new skills can significantly improve performance. During the chaos phase of the COVID-19 pandemic, enormous changes took place. Fear of COVID-19 infection and measures (physical distancing, curfews) changed the way of daily practice. eHealth solutions became unavailable, infrastructure collapsed, and feelings of anxiety, stress, and uncertainty about the future emerged. During this phase, we noticed that GPs took the initiative to create safe working conditions. They realized the new use of digital solutions and consulted via telemedicine. eHealth enabled them to reduce the burden on their daily practice work. They recovered their labor fees from the authorities and followed EU policies.

In the integration phase, integration occurs through the reinforcement of numerous practices and the new state of change. In the final stages of the COVID-19 pandemic, GPs routinely used teleservices as an integral part of their daily care to ensure continuity of care. They improved their digital capabilities through many apps. They integrated physical consultations and teleworking in this phase, allowing the mental shift to the new integration.

In the last phase, the new status quo, new working methods begin to be applied. New skills become second nature. New norms are formed as part of this. Work performance starts to align with the new skills, and a new status quo is formed. At this stage, we noticed that GPs were ready for unknown conditions and worked using the new digital services. They prioritized patient satisfaction. There was more sensitivity to ethical and privacy issues. Continuity of care has provided preparedness for future pandemics.

## Discussion

### Principal Findings

Our study indicates that the COVID-19 crisis was a critical turning point in adopting eHealth tools by GP practices in Flanders. Since the pandemic's start, the use of eHealth has rapidly increased and evolved. There was an increase in not only telemedicine but also mHealth, remote monitoring, and direct communication between health providers and patients [34].

According to the European Public Health Alliance (EPHA) briefing from November 2021, there has also been a tremendous increase in the deployment of digital health tools in European countries, such as Sweden and Italy, from doubling to a 30.1 times increase, respectively [42]. Tracking apps and Digital Green Certificates were developed to support the resumption of international mobility for tourism, leisure, and business, both internationally and in Europe.

GPs have been more flexible than ever before, working remotely, while maintaining continuity of care. Our respondents reported 3 factors facilitating eHealth uptake: COVID-19 pandemic measures, government and EU policies, and changing patient roles. Implementing teleservices for triage, charging for remote administration, and using digital applications (eg, COVID Safe, CoZo, and e-receipts). Interestingly, the GPs considered patient satisfaction with eHealth services essential in our study. This observation may support the hypothesis that the role of the patient has been actively changed, centered, and prioritized. During the pandemic, the participation of patients in eHealth use and teleservices was frequent, forcing the GPs to be more flexible with technological solutions. All these enablers have been documented in other studies.

This momentum was significant because health care was previously identified as 1 of the sectors lagging the furthest in adopting digital services. According to research, there were different reasons, such as the strict regulations of the sector, the sensitivity surrounding personally identifiable information, the resistance of health care providers to digital solutions, the lack of prioritization of patient experience, and the cost of investments [3-5]. Despite the potential benefits of eHealth in Belgium and other EU countries, uptake was slower than expected before the coronavirus crisis [28,43,44]. Research by the European Commission has shown that eHealth adoption in all European countries is much more complex and time-consuming than initially envisaged [45,46].

A Belgium study conducted between 2018 and 2019 identified infrastructure as the most significant barrier to eHealth adoption by GPs [27]. Respondents argued that although there had been improvement recently, there were continuity problems, crashes, and software updates when they were busy with patients and their electronic medical files. eHealth is seen as time-consuming, requiring significant investment and ICT services, leading to information overload, needing data security, and feeling dependent on external factors. However, the findings of this study differ from the previous research on eHealth perception and appreciation. Interestingly, there was a vast difference in the perception and appreciation of eHealth adoption before and after the COVID-19 crisis. In our study, the interviewed GPs agreed that the pandemic increased, boosted, and sped up their daily work, making eHealth essential. The GPs are aware of the benefits of eHealth and feel that eHealth is an important complementary part of health care, can reduce the burden of chaos, and increase access to health care. Contrary to what the earlier studies have found, the interviewees no longer see eHealth as a time-consuming tool; they see it as a time-saving instrument. All the GPs emphasized that eHealth is part of their work, stating that they will use it more in the future and noting that they are voluntarily investing in eHealth solutions.

In addition to the many advantages of eHealth use, the GPs noted some disadvantages, such as decreased privacy with the patients perceiving 24–7 online access to their doctor and increased busyness with the digital tools. In addition, their dissatisfaction was due to constantly being the digital administrator and prescribing more e-certificates than needed. These results have not previously been described in other studies.

One of the most important findings of our study is the mental shift of GPs to be more optimistic about teleworking. Although all the participants stated that physical contact with the patient would never lose its importance, the interviewees have become accustomed to working remotely and routinely practicing telemedicine. Telehealth is an appropriate and satisfactory modality for receiving care. It was essential to providing remote medical assistance, reducing GP workload, and helping increase patient satisfaction. This shift in mindset is evident because some GPs have stated that there will never again be only physical examinations and that a rational combination of telemedicine and physical examinations is vital. The interviewees also mentioned that the reimbursement conditions for telemedicine should be improved and continued. After the coronavirus crisis, teleconsultation became a complementary tool in the daily practice of Flemish GPs. These findings are similar to the results of previous quantitative and qualitative studies regarding the acceptance of eHealth during the COVID-19 pandemic [47,48].

In line with other studies [27,49], our study exposes patient digital literacy and the ICT burden as the main barriers to eHealth use. These are the primary obstacles noted by the GPs. The GPs stated that digital literacy is crucial for the elderly and socially deprived groups. They pointed to harmful discrimination perpetrated against these groups if only eHealth solutions were used. Further, blocking software and system crashes during work adversely affected eHealth uptake. Although the interviewees are satisfied with the massive development in IT solutions, more work still needs to be done. The GPs expect a stable and effective ICT health care strategy in the future. Confidentiality and security of patient data are no longer barriers to using eHealth but remain a concern among GPs. Other areas of concern include losing physical contact with the patient and missing follow-ups.

It is, therefore, likely that eHealth has become part of the daily routine in GP practices as a result of the pandemic. The practices demand a more stable IT infrastructure in the future to work efficiently, and more care communications between the first and second lines within the eHealth services are needed. This interorganizational coordination is crucial in order to create integrated services.

Finally, as eHealth becomes more implemented, we find that GPs are more open and flexible to using eHealth solutions, especially after the advent of COVID-19, and they feel more prepared for uncertain conditions, such as a pandemic. GPs’ digital literacy increased, and they intend to use more eHealth solutions, such as video consultations and mHealth apps, in the future.

### Limitations

There were some limitations to this study. First, practices in only a few districts in the Flemish region of Belgium with Dutch-speaking GPs were surveyed, and the experiences of other GPs in different regions were not considered. The sample size was quite small, and the use of the snowball method could have led to overrepresentation of GPs more “enthusiastic” toward eHealth. Nevertheless, our GPs also clearly indicated the risks, dangers, and drawbacks of eHealth and telemedicine. Further, the interviews were conducted between October 2021 and April 2022, and the GPs were asked about their experience of eHealth use since March 2020, when the COVID-19 pandemic started. This might have led to recall bias. In contrast, interviews were conducted in a period that eHealth use became already more integrated as a relatively sustainable routine in daily practice. As a result, eHealth use and the intention to continue using it may have changed marginally between the October 2021 and April 2022 interviews due to the normalization of GPs' daily practices.

Furthermore, we did not interview patients or other health care providers for our study. Therefore, our findings are limited to what the doctors detailed. In addition, we used semistructured interviews, which are highly accepted in terms of deep discussion, adaptability, locking into productive nature, empowering modern thoughts, and seeing interviews taken in their natural forms by counting nonverbal communication. However, we used Zoom to conduct online interviews due to the COVID-19 crisis, which could have resulted in a loss of information because the conditions are not the same as face-to-face interviews.

Further research will be beneficial for understanding which eHealth solutions could be implemented in the future, such as video consultations and mHealth apps, and their remuneration possibilities. Another important research topic is how to boost digital literacy for doctors and patients to sustain eHealth in the future. Finally, our qualitative research suggests that eHealth services have been adopted by GPs, but this hypothesis as well as the reach of implementation should be tested by quantitative research methods.

We recommend that health IT policymakers and developers maintain the continuity of eHealth solutions beyond the COVID-19 pandemic, considering the expectations and vulnerabilities presented in this study.

### Conclusion

With this study presented in the Flemish community of Belgium, we tried to acquire deep insights from the GPs into the perceived effects of the COVID-19 crisis on eHealth use and why and how things changed in their daily practices.

Previously defined areas of research were thoroughly analyzed, and we showed that the COVID-19 pandemic was a critical situation that provided significant and unstoppable changes in the uptake of eHealth by Flemish GPs. The coronavirus crisis was an accelerator for digital health care, which was previously described as ”backward.“ It made a positive mental shift toward eHealth adoption.

According to GPs, eHealth became essential to their daily practice during the COVID-19 crisis, reducing the burden on health care services and increasing health care accessibility. The perception of and appreciation for eHealth have changed positively, and eHealth has become an integral part of daily care. Patient satisfaction has increased and been prioritized significantly, and GPs have become more open and ready to implement more eHealth and mHealth solutions in their daily practice.

Although there has been a positive cognitive shift toward eHealth adoption, this study shows that there is still a significant amount of skepticism and uncertainty around privacy, security of patient data, digital literacy, and remuneration.

Future expectations were addressed as more integrated first and second services lines, a more stable IT infrastructure, and remuneration for digital services.

## Appendix

Multimedia Appendix 1 Interview guide.

## Abbreviations

AI: artificial intelligence
CoZo: Collaborative Care Platform
EU: European Union
GP: general practitioner
ICT: information and communication technologies
mHealth: mobile health
