# Supplementary material for: The Impact of the COVID-19 Pandemic on eHealth Use in the Daily Practice and Life of Dutch-Speaking General Practitioners in Belgium: Qualitative Study With Semistructured Interviews
Source: JMIR Form Res. 2022 Nov 28;6(11):e41847. doi: 10.2196/41847 (PMC9707615; doi:10.2196/41847)
Supplement: Multimedia Appendix 1 [file formative_v6i11e41847_app1.docx]

**Annex**

**Interview Guide Translated in English**

**Introduction**

Dear participant Dr. ,

First of all I would like to thank you for giving me this wonderful opportunity to interview you. My name is Ismail and I am a physician and also a student at the KU Leuven and I am following a master program 'The Management and Policy of Healthcare'. I am wondering about the impact of the COVID-19 pandemic on the daily use of digital technologies in general practice and by talking to you, I can better understand that. So I really appreciate you taking the time to talk to me.

In this interview, I'd like to dig deeper into your experience with digital health technologies in your practice during and after the COVID pandemic. So of course there are no right or wrong answers. It also means that you are welcome to tell us if there is anything that needs to be improved.

The interview will take no longer than half an hour, depending on how much we talk. And to get the formalities out of the way, I want to tell you that this interview is voluntary and you can opt out at any time and for any reason. I also want to tell you that everything you say is anonymized; So if I share some quotes from the interview with my colleagues, they will not know who said them.

Finally, I want to ask if I can record the audio of our interview? I am not going to share the recording with anyone. It will only help me focus on what you are saying if I don't have to take all my notes as we talk. You have my guarantee that I will delete the recording when I am done taking notes.

My interview will go like this;

- General features.

- Your daily routine

- Your use of digital technologies

- Your basic goals, motivations, experiences and opinions

- Ending with thanks and statement about data use

**General Characteristics**

1. May I ask what your age is ?

2. In which province do you work?

4. How many years have you been active as a general practitioner?

5. What type of practice do you currently work in and how many days a week do you work?

6. Can you describe to me what your daily work looks like please?

**Middle**

**Digital Technologies**

7. What do you think of when you hear the term "eHealth"?

[If interviewee does not know well what this is, please explain, also explain the different services by interviewer]

Follow-up question ; How do you appreciate the digitalization of your practice?

8. What is your view on the use of digital health technologies?

And more specifically by yourself?

9. Can you describe how you used digital health technologies for the COVID- 19 pandemic? Which of these are you already using? To what extent?

10. Can you describe what impact the COVID-19 pandemic has had on your work?

Follow-up questions; Have you used digital health technologies differently because of the pandemic? How? Try to ask all different aspects such as

Other services? Different use? New use and to what extent, More intensive use or not.... Only for you or in cooperation.

11. What your position on digital consultations? How does this work? How do you use this? What is your experience with it?

Do you have any specific examples? Communication, experiences, ..........

12. There are digital tools for patient follow-up. What do you think about them? Do you have any experience with them?

Follow-up question > What advantages do you see in this change?

Follow-up question > What are the main drawbacks?

Patient participation/doctor-patient relationship

13. In your opinion, how does digital consulting affect your interaction with patients?

And on patient ownership? >> What do you think are the advantages of digital consultations? And what are the disadvantages?

14. Can you say if how digital consulting has changed your communication with patients?

Future

15.What technologies would you like to continue or expand on in your practice?

Why?

16.What do you think it would take to incorporate these technologies into your practice?

17. How would you like to be assisted in doing so? ( What should be further developed to help you in your daily work?)

**End**

We are now at the end of the interview and I would like to conclude here. But before I conclude, could I ask you if there is anything you would like to add?

If so, I would like to thank you very much for talking with me. If you think of anything else or have any questions, please feel free to contact me. I also want to ask if I can contact you again if I think of other questions or if something is not clear.

Here's what I'm going to do with the data: I'm going to process it and summarize it. If you want, I can send it to you for review. If you want to suggest someone else to speak with, I can now get their contact information. It is also indeed good if you would like to ask if the other respondent would be willing to open the door with a phone call from you.

Have a nice day.

Jan Ismail Yagiz
